# Timeliness and completeness of weekly surveillance data reporting on epidemic prone diseases in Uganda, 2020–2021

**DOI:** 10.1186/s12889-023-15534-w

**Published:** 2023-04-04

**Authors:** Hildah Tendo Nansikombi, Benon Kwesiga, Freda L. Aceng, Alex R. Ario, Lilian Bulage, Emma S. Arinaitwe

**Affiliations:** 1 Uganda Public Health Fellowship Program, National Institute of Public Health, Kampala, Uganda; 2 Ministry of Health, Kampala, Uganda

**Keywords:** Disease surveillance, Epidemic Prone Diseases, Weekly Surveillance Data Reporting, Completeness, Timeliness, Uganda

## Abstract

**Introduction:**

Disease surveillance provides vital data for disease prevention and control programs. Incomplete and untimely data are common challenges in planning, monitoring, and evaluation of health sector performance, and health service delivery. Weekly surveillance data are sent from health facilities using mobile tracking (mTRAC) program, and synchronized into the District Health Information Software version 2 (DHIS2). The data are then merged into district, regional, and national level datasets. We described the completeness and timeliness of weekly surveillance data reporting on epidemic prone diseases in Uganda, 2020–2021.

**Methods:**

We abstracted data on completeness and timeliness of weekly reporting of epidemic-prone diseases from 146 districts of Uganda from the DHIS2.Timeliness is the proportion of all expected weekly reports that were submitted to DHIS2 by 12:00pm Monday of the following week. Completeness is the proportion of all expected weekly reports that were completely filled and submitted to DHIS2 by 12:00pm Wednesday of the following week. We determined the proportions and trends of completeness and timeliness of reporting at national level by year, health region, district, health facility level, and facility ownership.

**Results:**

National average reporting timeliness and completeness was 44% and 70% in 2020, and 49% and 75% in 2021. Eight of the 15 health regions achieved the target for completeness of ≥ 80%; Lango attained the highest (93%) in 2020, and Karamoja attained 96% in 2021. None of the regions achieved the timeliness target of ≥ 80% in either 2020 or 2021. Kampala District had the lowest completeness (38% and 32% in 2020 and 2021, respectively) and the lowest timeliness (19% in both 2020 and 2021). Referral hospitals and private owned health facilities did not attain any of the targets, and had the poorest reporting rates throughout 2020 and 2021.

**Conclusion:**

Weekly surveillance reporting on epidemic prone diseases improved modestly over time, but timeliness of reporting was poor. Further investigations to identify barriers to reporting timeliness for surveillance data are needed to address the variations in reporting.

## Background

The World Health Organization (WHO) launched the Integrated Disease Surveillance and Response (IDSR) strategy in African countries in 1998 [1, 2]. One of the main goals of IDSR implementation is to monitor disease and public health event trends in order to ensure that any unusual patterns such as outbreaks are detected quickly, investigated, and responded to within the shortest time [2–5]. Infectious disease outbreaks if not detected and reported early can rapidly spread and result in high morbidity and mortality [6]. To curb the effects of disease outbreaks, effective public health surveillance systems are needed to provide timely and accurate information leading to early detection of potential outbreaks and containing them in the local areas [5, 7].

The IDSR system in Uganda considers reportable priority diseases as per the third edition of IDSR Technical Guidelines launched in 2021 [8]. The diseases are categorized as follows: diseases targeted for elimination, epidemic prone diseases, diseases of public health importance and public health events of international concern under the International Health Regulations (IHR) of 2005 [8]. These priority diseases have varying reporting timelines and requirements [3, 8]. Surveillance data on these diseases are reported as immediate, weekly, monthly or quarterly reports; reports on epidemic prone diseases must be sent weekly [5, 8].

Diseases, conditions and events that are reported weekly include: Acute Flaccid Paralysis (AFP), Acute haemorrhagic fever syndrome (Ebola, Marburg, Lassa Fever, Crimean-Congo), Acute Jaundice, Adverse events following immunization (AEFI), Anthrax, Cholera, Dengue fever, Diarrhoea with blood (Shigellosis), Guinea Worm Disease (Dracunculiasis), Malaria, Malnutrition in under 5 years, Measles, Meningococcal Meningitis, Maternal death, Neonatal death, Neonatal tetanus, Plague, Rift Valley Fever, Severe Acute Respiratory Illness (SARI) clusters, Rabies, Typhoid, Yellow fever and laboratory confirmed multidrug and extremely drug resistant Tuberculosis [2, 8].

In Uganda, disease surveillance information is reported in a hierarchical order from the communities through health facilities using the short message service (SMS) 6767 platform and then to the Ministry of Health (MoH) reporting system [8]. At each level of reporting, the public health system as well as the technology involved contribute to problems of completeness, timeliness and data quality, leading to unreliable information for planning, monitoring and health service delivery [9, 10]. These challenges include: poor communication systems, inadequate financial resources, poor coordination, erratic feedback, inadequate training of health workers, poor supervision and mentorship, lack of IDSR technical guidelines, and reporting tools [11–14]. Each of these challenges, or in combination hinder countries from achieving optimal targets for IDSR implementation including timeliness and completeness [15]. To combat such challenges, surveillance systems need to be periodically assessed on key indicators such as completeness and timeliness of reporting to ensure that the objectives of surveillance are being met [8, 16]. For this reason, IDSR performance is often evaluated on completeness and timeliness of reporting through the District Health Information Software version 2 (DHIS2) based on a target of 80% reports being complete and timely [1, 15]. The DHIS2 automatically determines the number of reports submitted against the number expected to estimate completeness (by midday every Wednesday). It also indicates the number of reports which are submitted on time (by midday every Monday) [8].

After several years of IDSR implementation in Uganda, assessment of its performance was conducted in 2016 and revealed improvements in both timeliness (40–68%) and completeness (56–78%) of reporting at national level since 2012 when the second edition of IDSR was launched [4]. However, this assessment was conducted in only a few selected districts using district data. In addition, the DHIS2 system upgrade in 2019 might have impacted the performance of the surveillance reporting hence the need to conduct another assessment. However, DHIS2 data on completeness and timeliness of reporting were available starting from 2020 after a change in systems from the old to new DHIS2. We described the timeliness and completeness of weekly surveillance data reporting on epidemic prone diseases in Uganda, 2020 to 2021.

## Methods

### Study setting

Uganda is established under unique administrative units: health regions, districts, and health sub-districts [17]. By 2020, there were 15 health regions, 146 districts and 6,973 health facilities (3,133 public and 3,804 private-owned) [18]. Health service delivery is organised in tiers; from Health Centre (HC) I, HC II, HC III, HC IV, general hospital, regional referral hospital, and national referral hospital. Operationally, HC I are Village Health Teams (VHTs) that provide referral services to the higher levels. Each of the facilities is required to provide surveillance reports on epidemic prone diseases on a weekly basis [8].

#### Disease surveillance reporting procedures

**Fig. 1:**
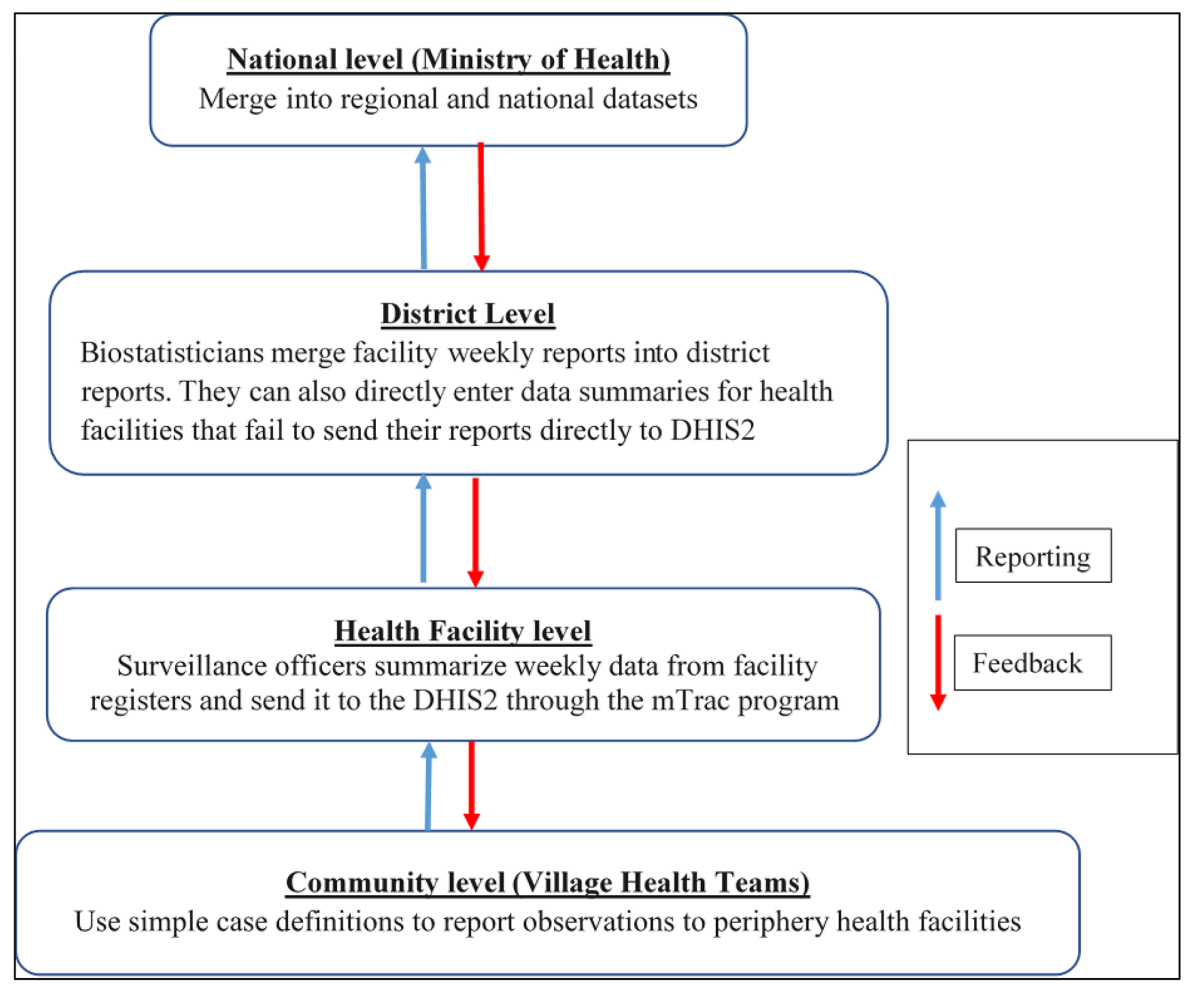
Reporting structure from the communities to the national level, Uganda

The DHIS2 is an open-source web-based platform maintained at the national level by the MoH. The software is used for collection, reporting, analysing, and disseminating health data as part of the Health Management Information System (HMIS) to inform planning and decision-making. DHIS2 was adopted at the national level in January 2011. The system was initially piloted in four districts, before it was rolled out to all the other districts by July 2012. As part of the roll-out process, 35 training workshops targeting 972 users were conducted throughout the country [10]. Subsequent revisions and trainings on the use of DHIS2 through the IDSR have been conducted since then [4, 19]. Disease surveillance reporting in Uganda follows a hierarchical order from community level to the national level of the health system through the DHIS2. At the community level, surveillance activities are conducted by community volunteers (village health teams) who are trained using simple case definitions and report their observations to the periphery health facilities. Then at the health facility level, the data are differentiated including information from out-patient, in-patient, consulting rooms and laboratory registers into summary sheets and IDSR reporting forms. The weekly epidemiological surveillance reports are then sent by facility surveillance officers through the 6767 SMS platform using mobile tracking (mTRAC) program, and synchronized into DHIS2. The data sent from health facilities to DHIS2 can then be merged into district, regional, and national level datasets (Fig.1). 

The DHIS2 system automatically determines the number of reports submitted against the number expected to estimate timeliness (by midday every Monday). It also indicates the number of complete reports (by midday every Wednesday) [8]. All levels of the health system are expected to meet the timelines for both timeliness and completeness at the same time. Each week, month, or quarter, a records clerk or statistician should summarize the proportions of reports received from their reporting sites for the different reporting periods. If any element of surveillance is being performed poorly at any level of the surveillance system, the higher level should provide support supervision to reinforce the opportunity for successful decision-making for improving and strengthening the surveillance system at the lower level. Supervisory visits are made to all surveillance sites under the poorly performing surveillance level [8].

### Study variables, data abstraction, and analysis

We analysed data on completeness and timeliness of weekly reporting of epidemic prone diseases from all the 146 districts of Uganda reporting through the DHIS2. Timeliness is the proportion of all expected weekly reports from the lower surveillance network to the next level using DHIS2 by 12:00pm of the Monday of the following week. Completeness is the proportion of all expected weekly reports from the lower surveillance network to the next level using DHIS2 by 12:00pm of the Wednesday of the following week. Timelines for all the levels of the surveillance system are the same. The DHIS2 has data on the total number of actual and expected reports for each week. For quality control, we selected a random sample of actual and expected number of reports. We randomly selected one district per epidemiological week of the study period, and compared the calculated proportions of timeliness and completeness of reporting with the DHIS2 computed proportions. All the calculated proportions were similar to the DHIS2 computed values. We determined the overall proportions and trends of completeness and timeliness of reporting at national level by year, health region, district, level of health facility, and health facility ownership. We analysed data using Epi Info version 7.

### Ethical considerations

We assessed routine surveillance data reported by districts to conduct an analysis of surveillance indicators, requested by the MoH. The data was aggregated with no identifying information. The US Centres for Disease Control and Prevention (CDC) provided non-research determination for this analysis since it wasn’t human subject research. We also obtained permission from MoH to use the data. All protocols for this project were reviewed in line with the US CDC policy by the US CDC human subjects’ review board in accordance with the declaration of Helsinki. All methods were carried out in accordance with the relevant guidelines and regulations.

## Results

### Timeliness of reporting weekly surveillance data on epidemic prone diseases, Uganda, 2020 − 2021

#### National trend of timeliness of reporting weekly surveillance data on epidemic prone diseases, Uganda, 2020 − 2021

**Fig. 2:**
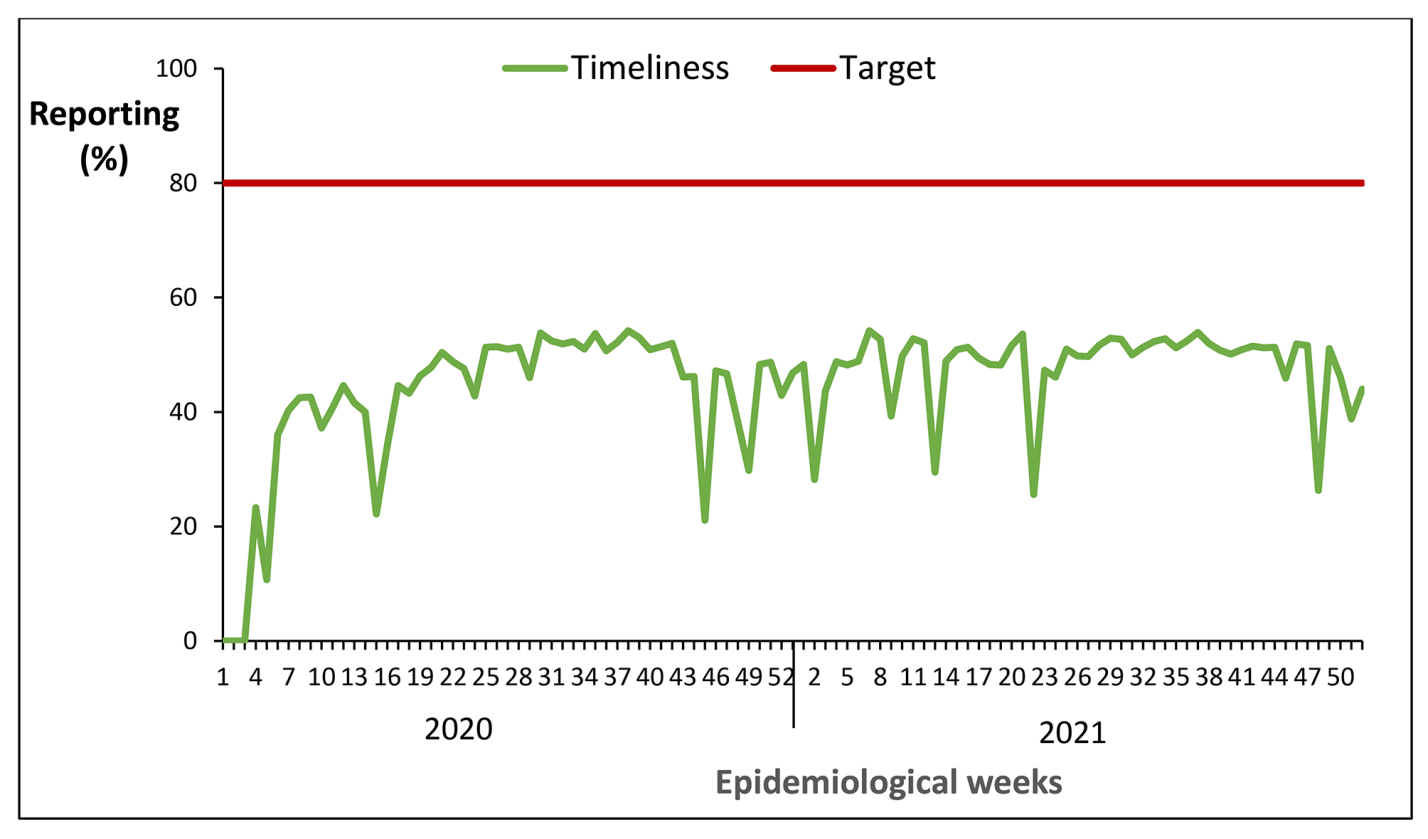
National timeliness of reporting weekly surveillance data on epidemic prone diseases, Uganda, 2020 − 2021

Timeliness of reporting was zero during the first three weeks of 2020 until epidemiological week four of 2020 with 23% timeliness of reporting. The national timeliness of reporting was below the 80% target throughout 2020 and 2021 (Fig. 2). The national timeliness of reporting was 49% in 2021 compared to 44% in 2020, indicating a 5% increase over the two-year period.

#### Timeliness of reporting weekly surveillance data on epidemic prone diseases by health region, Uganda, 2020 − 2021

**Fig. 3:**
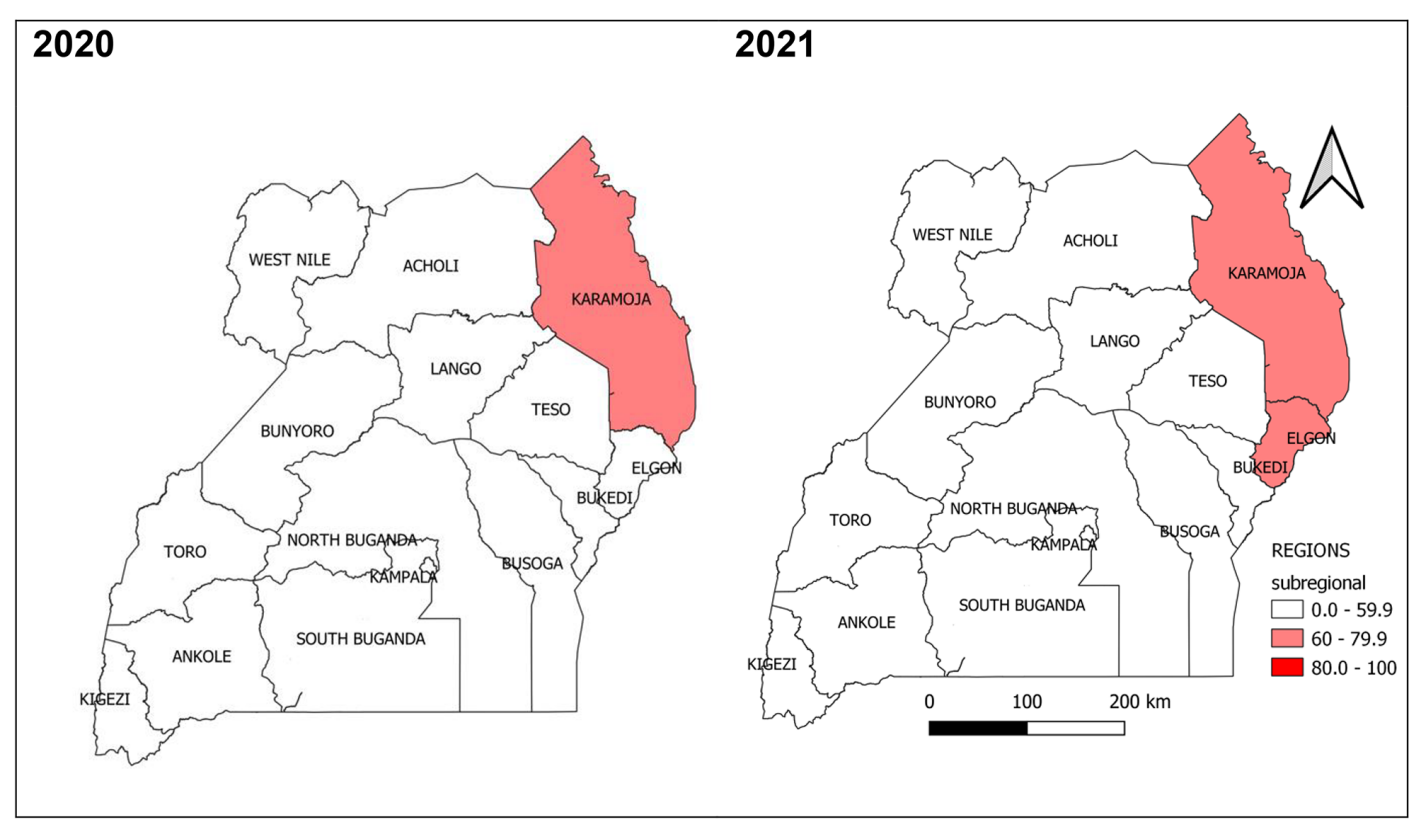
Timeliness of reporting weekly surveillance data on epidemic prone diseases by health region, Uganda, 2020–2021

None of the 15 health regions achieved the national target for timeliness of reporting at 80%. However, there was a notable increase in timeliness of reporting across all health regions except Kampala region which also attained the lowest in timeliness of reporting (19%) in both 2020 and 2021 (Fig. 3).

#### Timeliness of reporting weekly surveillance data on epidemic prone diseases by district, Uganda, 2020 − 2021

**Fig. 4:**
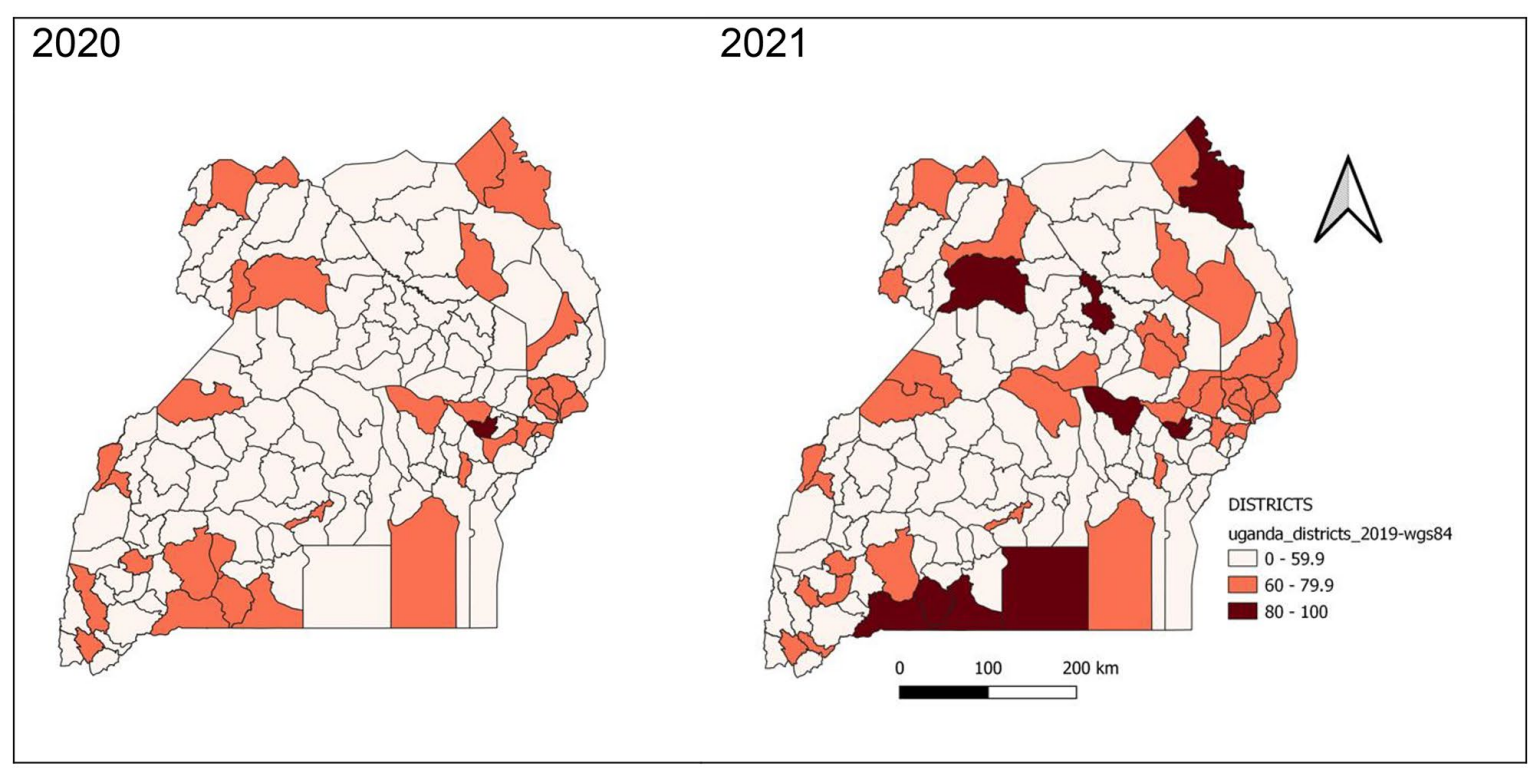
Timeliness of reporting weekly surveillance data on epidemic prone diseases by district, Uganda, 2020–2021

Timeliness of reporting was poor throughout 2020 and 2021, below 60% in many of the districts. Only Kibuku District attained the 80% target for timeliness of reporting in 2020 (81%). In 2021, nine districts improved and attained target for reporting timeliness: Buyende (88%), Isingiro (84%), Kibuku (83%), Rakai (82%), Nwoya (98%), Lira (80%), Kalangala (86%), Kyotera (86%), and Kaabong (82%) (Fig. 4).

#### Timeliness of reporting weekly surveillance data on epidemic prone diseases by level of health facility, Uganda, 2020 − 2021

**Fig. 5:**
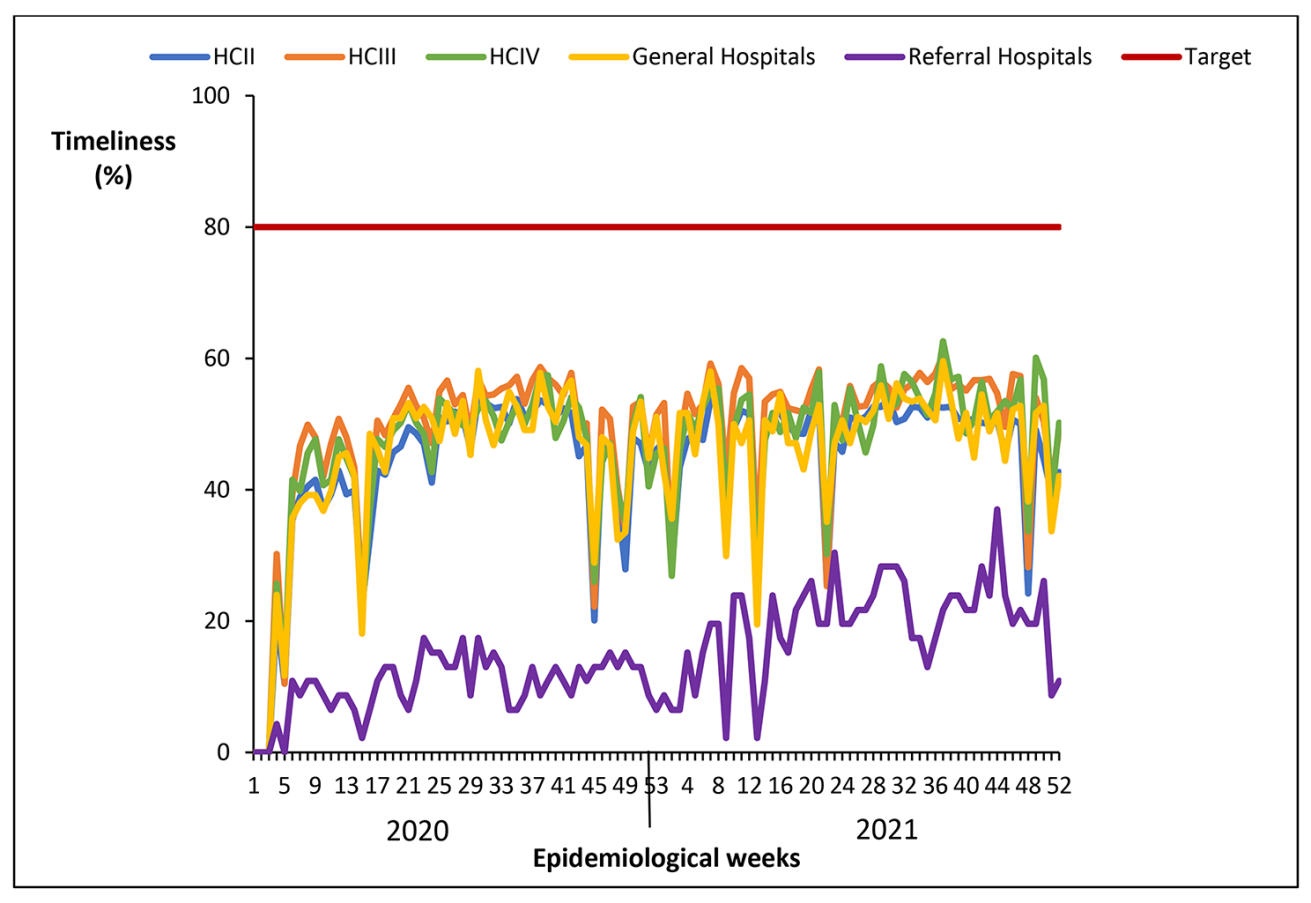
Timeliness of reporting weekly surveillance data on epidemic prone diseases by level of health facility, Uganda, 2020 − 2021

All health facilities were consistently below the 80% target for timeliness of reporting, and maintained a similar pattern throughout 2020–2021. Referral hospitals had the lowest reporting rates for timeliness compared to other levels of health facilities (Fig. 5).

#### Timeliness of reporting weekly surveillance data on epidemic prone diseases by health facility ownership, Uganda, 2020 − 2021

**Fig. 6:**
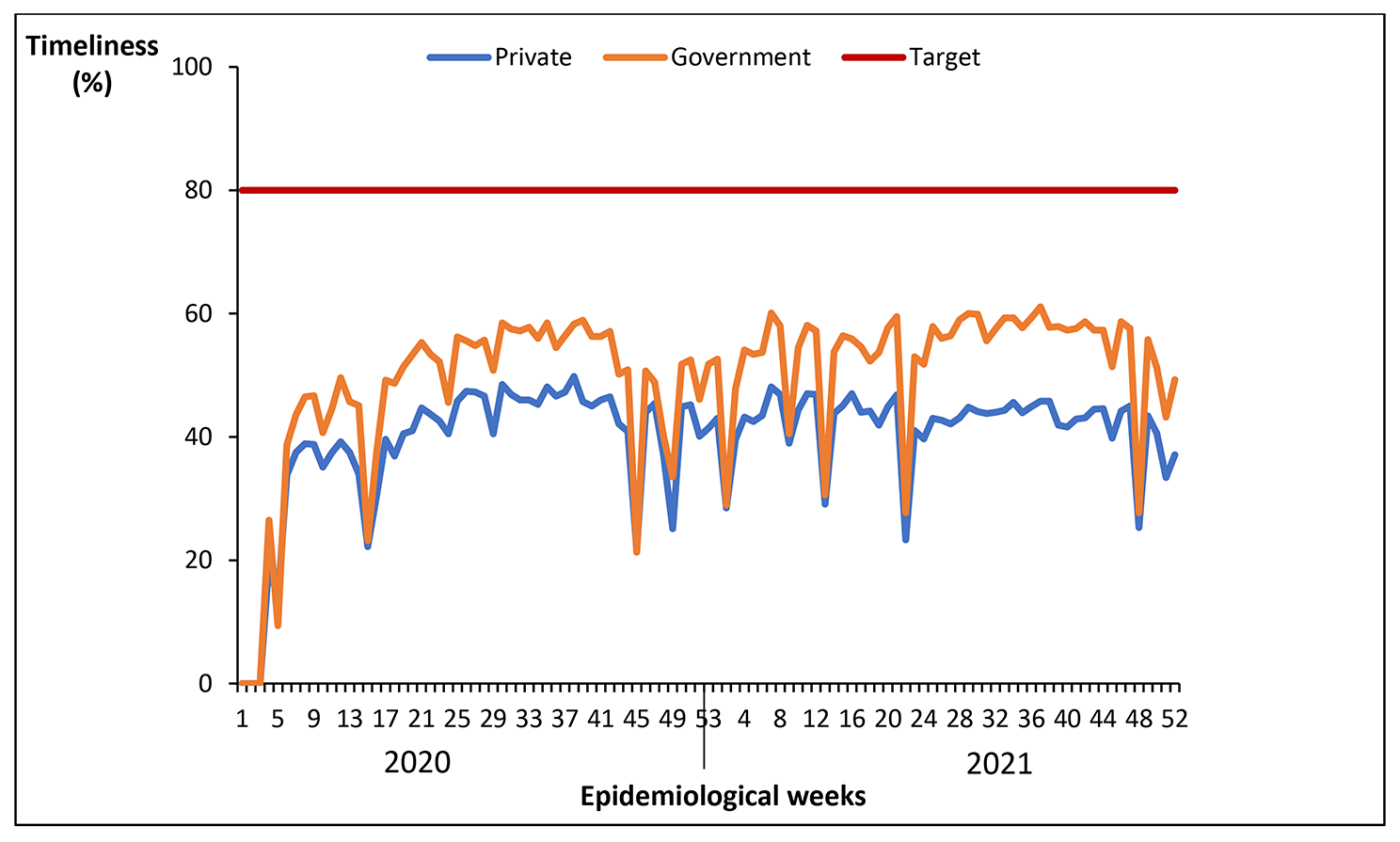
Timeliness of reporting weekly surveillance data on epidemic prone diseases by health facility ownership, Uganda, 2020 − 2021

Both government and private owned health facilities had a similar trend for timeliness of reporting. None of the facilities attained the 80% target for timeliness of reporting throughout 2020–2021. Private health facilities had lower rates of timeliness of reporting compared to government facilities (Fig. 6).

### Completeness of reporting weekly surveillance data on epidemic prone diseases, Uganda, 2020 − 2021

#### National trend of completeness of reporting weekly surveillance data on epidemic prone diseases, Uganda, 2020 − 2021

**Fig. 7:**
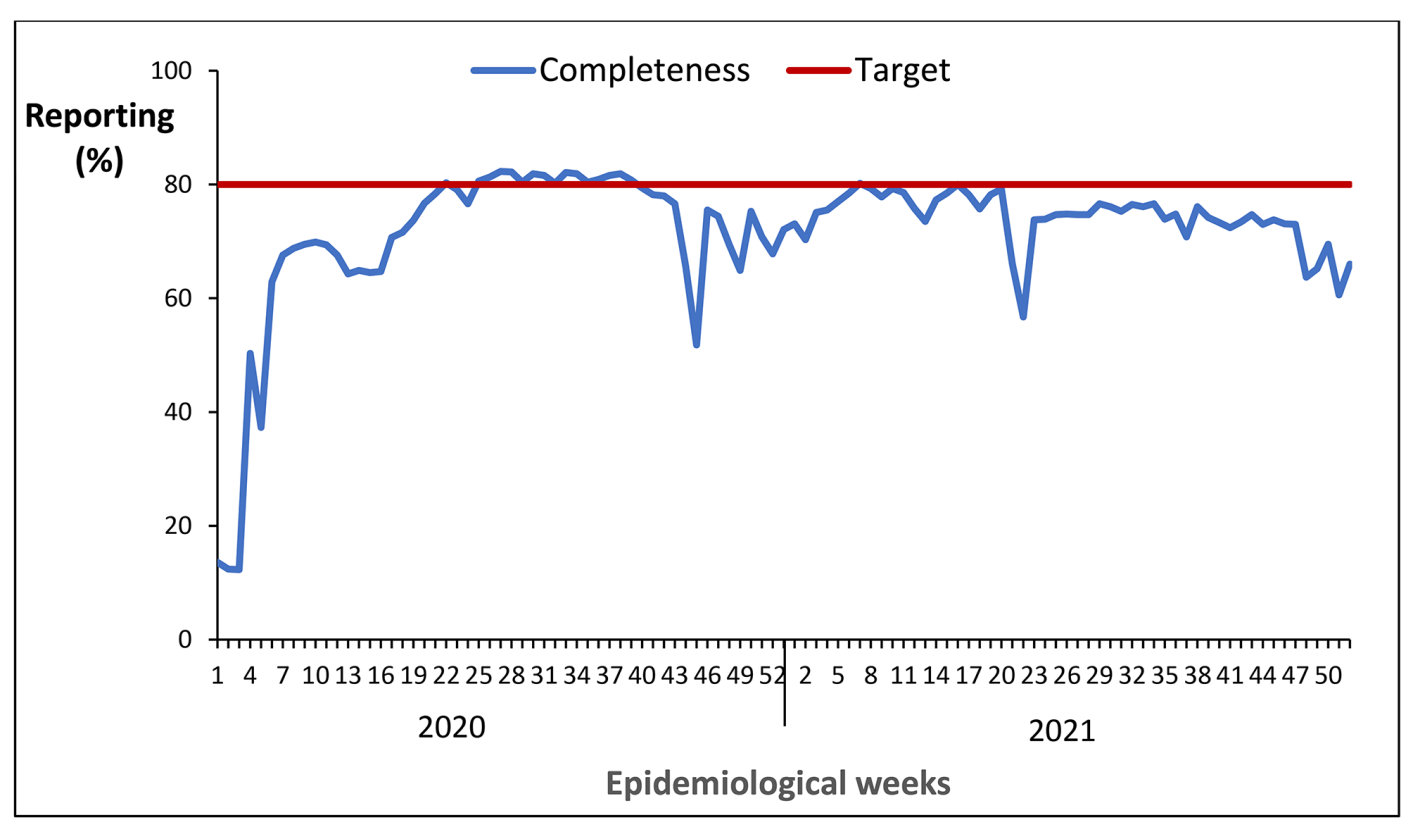
National completeness of reporting weekly surveillance data on epidemic prone diseases, Uganda, 2020 − 2021

The national completeness of reporting was 14% in epidemiological week one of 2020, increased over time and reached the 80% target at epidemiological week 22 of 2020 though dropped at week 43 and remained below the target until the end of 2021 (Fig. 7). The national completeness of reporting was 75% in 2021 compared to 70% in 2020, indicating a 5% increase over the two-year period.

#### Completeness of reporting weekly surveillance data on epidemic prone diseases by health region, Uganda, 2020 − 2021

**Fig. 8:**
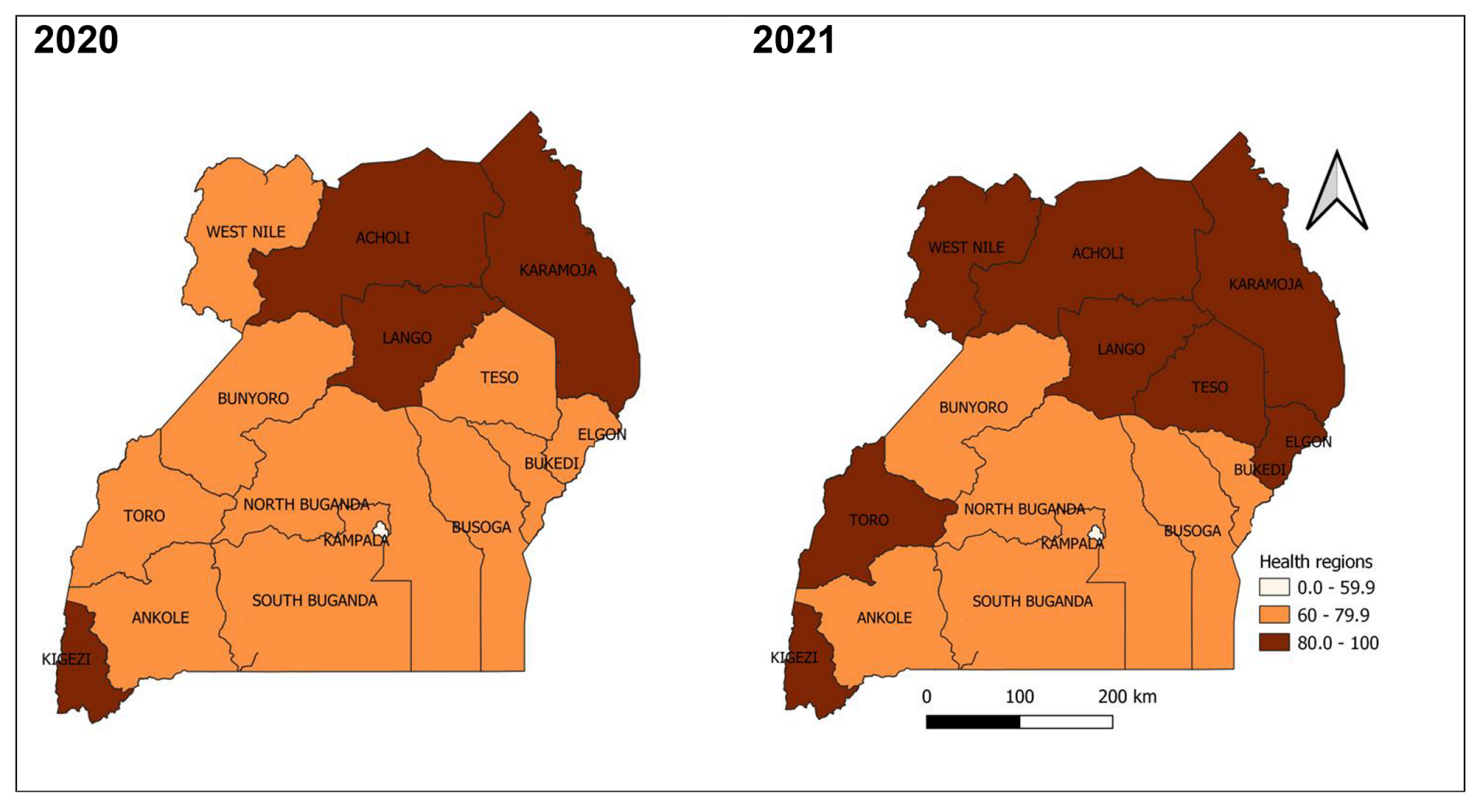
Completeness of reporting weekly surveillance data on epidemic prone diseases by health region, Uganda, 2020–2021

Of the 15 health regions, eight achieved the target for completeness of reporting at 80%; Karamoja and Lango Regions attained the highest 96% and 93% in 2021 and 2020 respectively. Unlike other regions registering improvement in completeness of reporting from 2020 to 2021, Kampala region attained the lowest and registered a 6% decrease: 38% and 32% in 2020 and 2021 respectively (Fig. 8).

#### Completeness of reporting weekly surveillance data on epidemic prone diseases by district, Uganda, 2020 − 2021

**Fig. 9:**
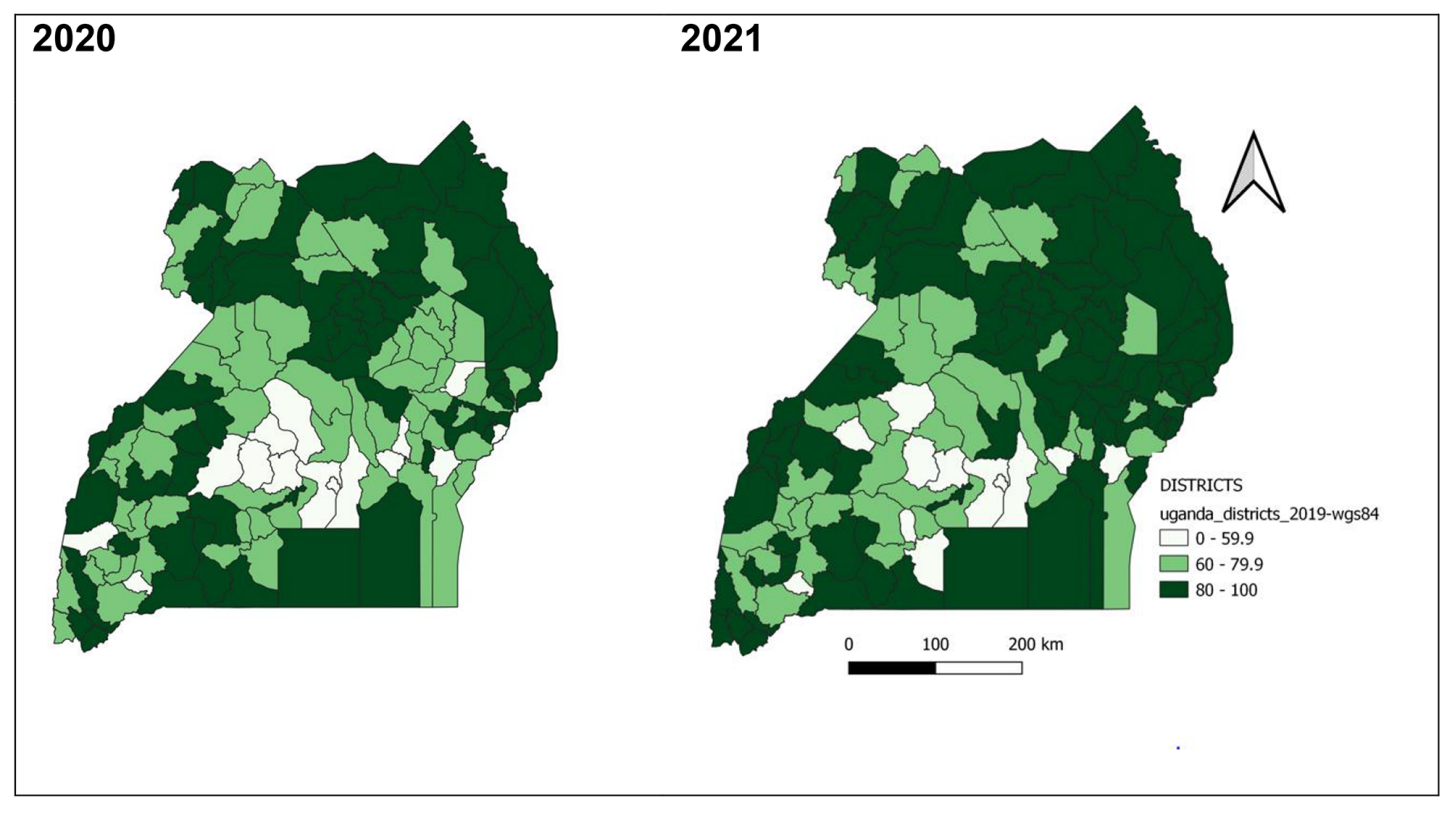
Completeness of weekly surveillance data reporting on epidemic prone diseases by district, Uganda, 2020–2021

Majority of the districts achieved the 80% target of completeness of reporting in 2020 and improvements continued to be seen in 2021. All districts in Karamoja region attained and maintained the 80% target of completeness throughout 2020 and 2021. Districts of Kampala, Busoga Region (Bugiri, Jinja), and South-central Region (Bukomansimbi, Masaka, Kassanda, Wakiso), and Rwampara continued to perform poorly with less than 60% completeness of reporting throughout 2020 and 2021 (Fig. 9).

#### Completeness of reporting weekly surveillance data on epidemic prone diseases by level of health facility, Uganda, 2020 − 2021

**Fig. 10:**
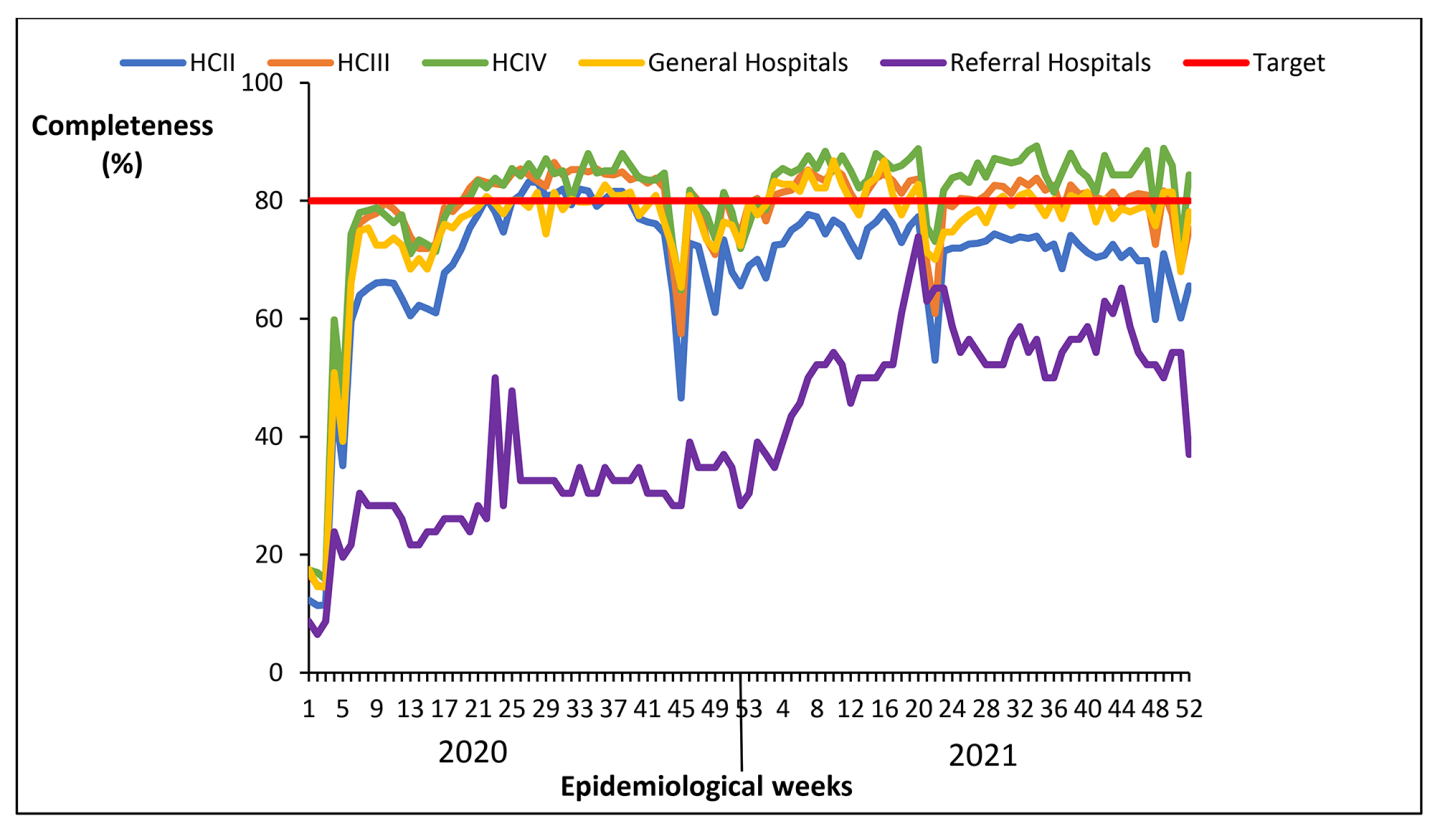
Completeness of reporting weekly surveillance data on epidemic prone diseases by level of health facility, Uganda, 2020 − 2021

Completeness of reporting by level of health facility improved over time from 2020 to 2021. The trend was similar across all health facility levels, with HCIII, HCIV and general hospitals attaining the 80% target. Regional referral hospitals and HCIIs had lower rates of completeness of reporting compared to other levels of health facilities (Fig. 10).

#### Completeness of reporting weekly surveillance data on epidemic prone diseases by health facility ownership, Uganda, 2020 − 2021

**Fig. 11:**
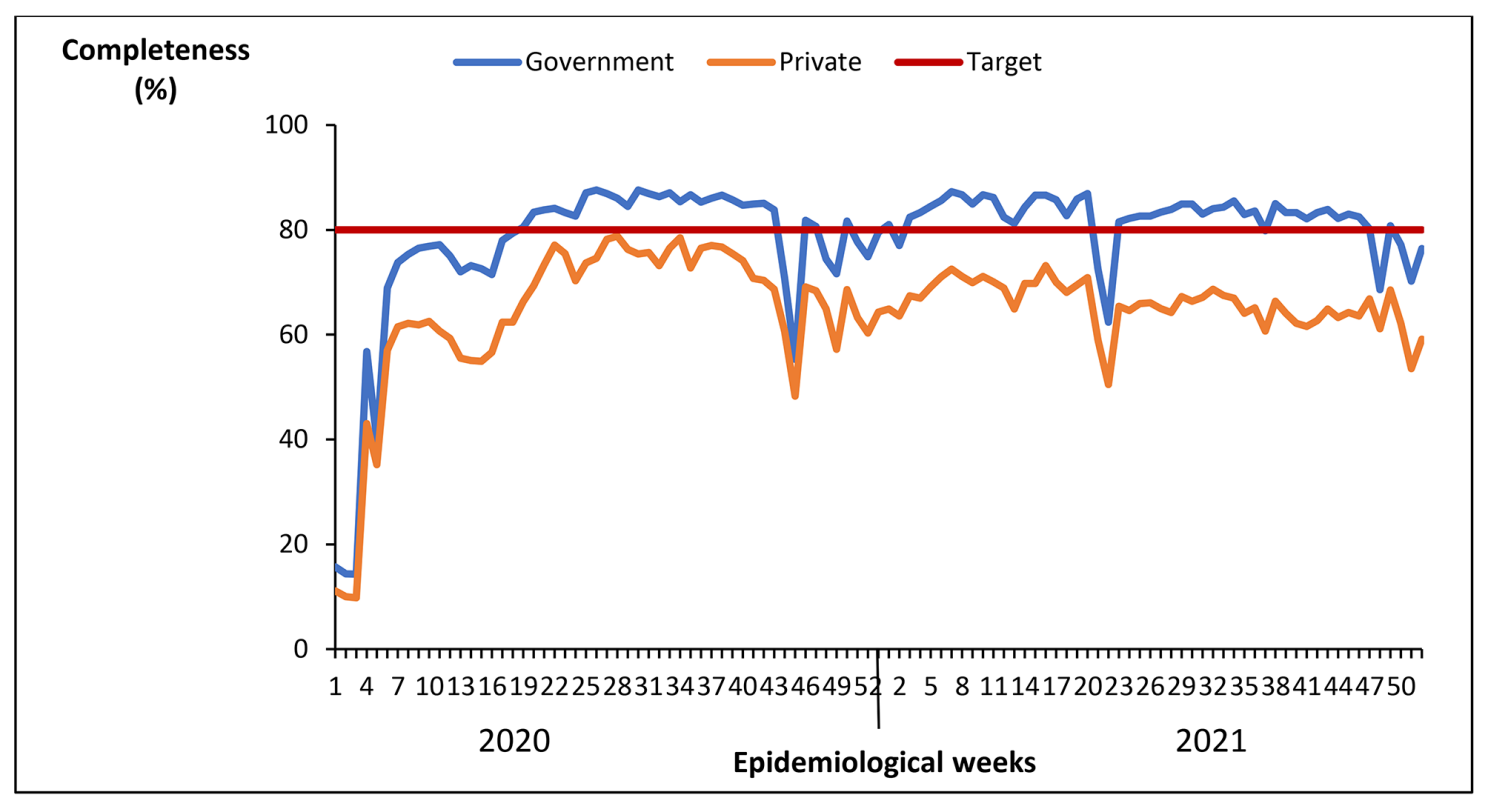
Completeness of reporting weekly surveillance data on epidemic prone diseases by health facility ownership, Uganda, 2020–2021

Only government owned health facilities reached the 80% target for completeness of reporting. Private health facilities were below the target throughout 2020–2021 but with a similar trend as the government facilities (Fig. 11).

## Discussion

This study addresses an important aspect of public health surveillance systems in Sub-Saharan Africa (SSA). Our findings indicate improvements in surveillance data reporting both at regional and national levels, which supports similar findings from SSA of progress in reporting completeness and timeliness associated with either IDSR system or DHIS2 implementation [5, 20, 21]. In spite of the observed improvements, the overall reporting completeness and timeliness remain insufficient below the 80% target, and vary greatly by health region, district, level of health facility, and health facility ownership.

Although Kiberu et al. argued that challenges of data reporting seem to have been resolved through the use of DHIS instead of paper-based forms in Uganda, this may have worked for a few districts [10]. The increases in both completeness and timeliness of reporting are likely due to the internet-based reporting and continuous reminders of reports submission through personal mobile phones as it has been reported from other African countries implementing IDSR [22, 23]. In addition, the ongoing COVID-19 pandemic might have increased biostatisticians’ and surveillance focal persons’ alertness and understanding of the need for surveillance data reporting, thus the improvement in reporting completeness and timeliness as reported by similar studies [24, 25]. However, our findings may not fully support this since data before the pandemic are not available for us to understand the impact of the pandemic on surveillance data reporting. On the other hand, the poor reporting rates in some districts might have been influenced by poor motivation, network and internet challenges, which have potential for error introduction thus affecting data accuracy [26]. Furthermore, the COVID-19 pandemic had substantial impact on health systems affecting health workers’ productivity with high rate of burn out, COVID-19 infection, deaths and psychological stress [27–29]. This might be linked to poor reporting rates in some areas.

The findings further revealed low and varied levels in the reporting timeliness at regional, district, and health facility levels. This is in line with previous studies conducted in Africa and America which reported that low timeliness is still common at all levels of health services [21, 30]. The possibility of missing outbreaks and delays in public health response such as contact tracing, due to untimely and incomplete reporting appear to be a real challenge in the Uganda health system.

At health facility level, lowest reporting rates for both completeness and timeliness were observed in referral hospitals. Surveillance officers responsible for reporting at referral hospital level are usually clinicians, who have high numbers of patients to attend to critical illnesses. These officers perform a variety of tasks in addition to those outlined in the job description resulting in issues of increased workload, and competing demands of task-shifting and prioritizing curative care versus surveillance reporting similar to findings in Madagascar [31]. Also, the low reporting rates observed in private health facilities might be attributed to high employee turn-over, or unavailability of trained personnel to compile and submit surveillance reports as similarly reported in other African countries [21, 31–34].

Reporting performance is often affected by circumstances where the person responsible for compilation and submission of the reports is too busy or unavailable; similar to findings in Kenya [32]. There is need to involve all health workers in IDSR training and impart knowledge on the reporting requirements across all surveillance levels [33]. In addition, integrating IDSR training in pre-service curricula for health training institutions might address the gap in sustainability of trained human resource [19]. In case the designated officer is unavailable, a colleague can be able to step in. Furthermore, enhanced and continued training of surveillance staff in addition to routine validation of data reports by biostatisticians can help improve completeness, timeliness and data quality of reporting [21, 31, 34]. However, the private sector may not make themselves available for such engagements due to “loss” of business when they are away at a training, and the lack of designated officers for surveillance data reporting [35]. Therefore, these findings suggest the need for further investigation to understand how to effectively involve the private sector in surveillance of epidemic prone diseases.

Our study should be interpreted based on the following limitations. Firstly, the findings were based on a short duration since data were only available in DHIS2 from 2020; the data only covered the COVID-19 pandemic period. During the last quarter of 2019, there was a system upgrade of the DHIS2. Users found it difficult to adapt and use the system hence the loss of some data during the last quarter of 2019 and first few weeks of 2020. Further, due to the same system upgrade, there was loss of some health facility data for the previous years. We couldn’t therefore describe reporting before and during the pandemic to establish its effect on surveillance data reporting. Secondly, common challenges with internet data transmission in all parts of Uganda might have introduced some data errors resulting in bias in our findings. Such network challenges deter transmission and synchronizing of reports into the DHIS2. Our findings might therefore underestimate timeliness and completeness of weekly epidemiological reports.

## Conclusion

Timeliness and completeness of weekly epidemic prone disease surveillance reporting through DHIS2 improved over time. However, despite these improvements, timeliness of reporting still remains poor below target in most of the districts and all health regions. We suggest continuous support supervision, mentorship and additional system/infrastructure enhancements, including internet connectivity, to further enhance surveillance data reporting. Further investigations to identify barriers to reporting completeness and timeliness of surveillance data are needed to address the variations in reporting rates.

## Data Availability

The data that support the findings of this investigation belong to the Uganda Public Health Fellowship Program. However, the data can be accessed upon reasonable request from the corresponding author and with permission from the Uganda Public Health Fellowship Program.

## List of Abbreviations

AEFI: Adverse Events Following Immunization
AFP: Acute Flaccid Paralysis
CDC: Centres for Disease Control and Prevention
DHIS2: District Health Information System version 2
DHO: District Health Office
HC: Health Centre
HMIS: Health Management Information System
IDSR: Integrated Disease Surveillance and Response
IHR: International Health Regulations
MoH: Ministry of Health
mTRAC: Mobile tracking
SARI: Severe Acute Respiratory Illness
SMS: Short Message Service
SSA: SubSaharan Africa
VHT: Village Health Team
WHO: World Health Organisation
